# Developing a model of recovery in mental health

**DOI:** 10.1186/1472-6963-9-73

**Published:** 2009-05-01

**Authors:** Sylvie Noiseux, Denise St-Cyr Tribble, Claude Leclerc, Nicole Ricard, Ellen Corin, Raymond Morissette, Roseline Lambert

**Affiliations:** 1 Faculté des Sciences infirmières, Université de Montréal, 2375 Chemin de la Côte Sainte-Catherine, C.P. 6128, succursale A, Montréal, Québec, H3C 3J7, Canada; 2 École des sciences infirmières, Faculté de médecine et des sciences de la santé, Université de Sherbrooke, 3001, 12e Avenue Nord, Sherbrooke, Québec, J1H 5N4, Canada; 3 Département des sciences infirmières, Université du Québec, à Trois-Rivières, 3351 Bd des Forges Trois-Rivières, Québec, G9A 5H7, Canada; 4 Institut Douglas Recherche, Pavillon Lehmann, bureau G-1123, 6875, Montréal boul. LaSalle, Verdun, Québec, H4H 1R3, Canada; 5 Hôpital Louis-H. Lafontaine, 7401, rue Hochelaga, Québec, H1N 3M5, Canada; 6 Centre de recherche Fernand Seguin, 7331, rue Hochelaga, Montréal (Québec), H1N 3V2, Canada

## Abstract

**Background:**

The recovery process is characterized by the interaction of a set of individual, environmental and organizational conditions common to different people suffering with a mental health problem. The fact that most of the studies have been working with schizophrenic patients we cannot extend what has been learned about the process of recovery to other types of mental problem. In the meantime, the prevalence of anxiety, affective and borderline personality disorders continues to increase, imposing a significant socioeconomic burden on the Canadian healthcare system and on the patients, their family and significant other [1]. The aim of this study is to put forward a theoretical model of the recovery process for people with mental health problem schizophrenic, affective, anxiety and borderline personality disorders, family members and a significant care provider.

**Method and design:**

To operationalize the study, a qualitative, inductive design was chosen. Qualitative research open the way to learning – the inside – about different perspectives and issues people face in their process of recovery. The study proposal is involving a multisite study that will be conducted in three different cities of the Province of Québec in Canada: Montréal, Québec and Trois-Rivières. The plan is to select 108 participants, divided into four comparison groups representing four types of mental health problem. Each comparison group (n = 27) will be made up of 9 units. Each unit will comprise one person with a mental health problem (schizophrenia, affective anxiety, and borderline personality disorders. Data will be collected through semi-structured open-ended interview. The in-depth qualitative analysis inspired from the grounded theory approach will permit the illustration of the recovery process.

**Discussion:**

The transformation of our Health Care System and the importance being put on the people well-being and autonomy development of the person who are suffering with mental problem This study protocol follows-up on earlier theory-building process that begun with the work of Noiseux [2]. The contribution of the present study is to increase the comprehension of the concept of recovery and to enhance the body of knowledge in that domain. Very few studies have examined recovery and the one that did used a descriptive approach which did not take into account the perspective of the family members and the caregivers of the recovery process.

## Background

The aim of this research project is to continue the work of theory-building begun in the study by Noiseux [2] and to put forward a substantive theoretical model of recovery for people with a mental health problem. The originality of Noiseux's [2] work rests in the fact that it proposes an explanation of the dynamics of the various mechanisms that come into play in the recovery of people with schizophrenia. Most particularly, the findings made it possible to identify empirical indicators that characterize the interaction between the individual, environmental and organizational conditions that influence the recovery process. The work thus constitutes a first step in the development of a substantive theoretical model and confirms the undeniable importance of broadening the explanation of recovery to include other groups of people with a mental health problem.

### Statement of the problem and relevance of the study

In Canada, recovery has become the guiding principle of the mental health system, resulting in advocacy for care and services that would facilitate the process [3] and eliciting a clear political will and great enthusiasm. However, although the literature regularly refers to a "model" of recovery, no such model exists; none has actually been defined and implemented in Canada [3]. Moreover, recovery assumes numerous meanings, depending on the context in which it is raised, and the notion may create considerable confusion among people with a mental health problem and their family as well as among clinicians, policy makers and researchers [4]. Indeed, recovery is often confused with the concepts of remission, cure, readjustment, and even rehabilitation [5]. Consequently, the meaning of the recovery process has lost any specificity, and there are all too real risks of a reductive understanding of the concept in the implementation of services.

Over the past twenty years, many research studies have described the nature of recovery and some of the internal and external conditions associated with it and have thus contributed to the development of new knowledge about the process. The literature indicates that recovery is not a cure, but a profoundly personal path that individuals may follow; it entails work, particularly work on themselves, their feelings, desires, competencies, roles, and plans for the future [6,7]. These internal conditions are thus related to an individual's attitudes and processes of change. The external conditions involve the interpersonal environment, the organization of services and politics. In general, the studies bring out four central conditions for recovery: (a) acknowledgment of the mental disorder, (b) transformation of the sense of self, (c) reconciliation with the system, and (d) the development of interpersonal relations [8].

Although these studies have helped identify conditions associated with recovery, they have not provided an explanation of how they influence each other or of how the mechanisms by which they operate might help us understand the process through which people manage to recover. It is thus important to recognize that because of the limitations of all the studies up to now, it has not been possible to posit a theoretical model of recovery. These limitations stem mainly from the research methods adopted and from the use of limited samples drawn from a single data source (e.g., people with mental health problems). The studies have primarily used exploratory and descriptive study designs and were conducted with people who had been diagnosed with schizophrenia. They were not extended to include a variety of data sources that would have enabled the investigators to take into account the perceptions of other actors, such as family members and care providers, who are directly concerned with the recovery process [9-16]. Furthermore, the concept of recovery is most often used for all serious mental health problems without distinction. Does that mean that the process is the same for people with different types of mental health problems? Given the limitations of the papers we have reviewed, we cannot answer this question. It is thus clearly appropriate to broaden the study of the recovery process to include groups of people with other types of mental health problems, such as affective, anxiety or even borderline personality disorders. Mental health problems do, in fact, present certain similarities, notably on the level of the progression of the disease. However, they differ in terms of the nature of the symptoms and their impact on biopsychosocial functioning. It is therefore important to identify the similarities and differences that characterize recovery in terms of specific mental health problems. It would thus be possible to develop a substantive theoretical model whose parameters could be generalized but still take into account the particular traits of individuals and their health condition.

Furthermore, developing a theoretical model of recovery involves a new paradigm, whose scope cannot be considered unless a wide range of perspectives from different data sources are taken into account [17]. Noiseux's [2] findings thus demonstrate the appropriateness of including people with a mental health problem, family members and care providers to buttress and refine the development of a theoretical model of recovery. The perceptions of family members can make a great contribution to increasing our understanding of the recovery process since they have, for the most part, known the individuals affected since before the onset of the disease; they have followed their progress closely and often been witness to their regression or positive evolution. The care providers are also an important source of information because of their role in raising people's awareness of their resources and potential. In fact, care providers often witness significant outcomes in the condition of such individuals that go far beyond notions of stabilization or improvement. Up to now care providers have not been questioned much in studies on recovery, apart from a few in which the investigators attempted to verify how the recent implementation of intervention programs in the community may have fostered recovery [18-20].

In general, the studies have significant limitations that imposing consequences for the interpretation of the recovery process and the implementation of mental health services and care. In the absence of a sound theoretical model, a linear vision of recovery currently guides mental health programs and services; they advocate that the individual follow a series of predetermined steps based on notions of normalization to attain structured objectives [21,22]. Mental health services also make many efforts to put into place external conditions for recovery, such as access to housing or even to sheltered employment. Though necessary, such conditions have been shown to be clearly insufficient because recovery is primarily a profoundly personal process that cannot be activated or initiated solely by external factors [23,24]. In other words, while some healthcare systems seek to organize modes of practice in terms of principles related to recovery, it has nonetheless been difficult to put into place a dynamic conception of recovery that takes into account the interplay of individual, environmental and organizational conditions [3,24,25]. It is therefore important to carry on this process of expanding and refining the model of recovery begun by Noiseux [2], taking into consideration the findings that emerge from earlier studies and current practices in mental health. Considering the phenomenon under investigation, the grounded theory approach [26] seems entirely appropriate. This approach allows for development of a substantive theoretical model; that is, one that relates to the practical dimension of situations [27]. Grounded theory calls for the use of a variety of data sources to better understand, delineate and explain the phenomenon under investigation. At the same time, it requires pushing beyond descriptive analysis by defining concepts or categories and relating them to each other in a model that comprises a refined, detailed proposal [28].

### Purpose and objectives of the study

The purpose of this study is to continue the theory building begun by Noiseux [2] and to put forward a substantive theoretical model of recovery for people with a mental health problem. The study comprises three specific objective which are to: 1) deepen understanding of the recovery process among four groups of people with different types of mental health problem; namely, people with (a) schizophrenic disorder, (b) affective disorder, (c) anxiety disorder, and (d) borderline personality disorder; 2) analyze the similarities and differences that characterize the recovery process for people in each comparison group; 3) validate the empirical indicators of the recovery process.

### Review of the relevant literature

#### Prevalence and impacts of mental health problems

In 2001, the World Health Organization (WHO) reported an increase in mental health problems around the world, while observing a decline in the prevalence and incidence of physical health problems [29]. In Canada, the Canadian Community Health Survey, Statistics Canada [30] estimates the overall prevalence of mental health problems at 10% to 11%, depending on gender. The most common problems are related to anxiety disorders (7% and 15%) and depressive disorders (4.5%). It is estimated that serious mental disorders, such as schizophrenia, generally involve 1% to 3% of the population [31]; borderline personality disorders are said to affect 2% [32]. The impact of these mental health problems on individuals, their family and society is enormous. It is estimated that depression, bipolar disorders and schizophrenia rank in the top five in terms of social and family costs. What is more, according to the Canadian Mental Health Association, the effects of a diagnosis of mental disorder–including, among other things, iatrogenesis (adverse effects stemming from psychiatric treatment), the designation of a handicap, despair, and stigmatization–are as devastating as the disease itself [3]. Profound changes in mental health policies and services must be implemented to reduce these extremely harmful effects. Recovery has thus become the guiding principle of the mental health system in Canada; it appears as a new paradigm requiring further development, especially for people suffering from schizophrenia, bipolar disorders, depression, and borderline personality disorder [3]. The rediscovery of recovery is a hopeful development insofar as the traditional conception of the phenomenon, which is generally associated with an observable outcome or a cure, is put into perspective through research conducted on sound theoretical bases [2].

#### Rediscovery of the phenomenon of recovery

The review of the literature reveals four main types of studies that have contributed to the emergence of a new perspective on recovery in mental health. The research may be grouped into (a) longitudinal studies, (b) autobiographical and anecdotal accounts, (c) qualitative studies, and (d) studies of models of recovery-oriented services. Longitudinal studies conducted since the 1980s have been chiefly behind the emergence of the hypothesis of possible recovery for people with a mental disorder, associating the phenomenon with functional improvement in health status [33-41]. Although these studies open the way to cultivating new knowledge about recovery, it must be emphasized that they are primarily concerned with the progression of the disease and so do not really make it possible to advance our understanding of the phenomenon. Recovery is dealt with mainly as a return of functional capabilities or of functioning in activities of daily life [42-45].

As the autobiographical accounts of people with a mental health problem [46-50] and the theoretical writings of key authors [6,19,51-56] point out, how a person's health evolves is more than a matter of the restoration of functional capabilities. Indeed, these works highlight the notions of process and experience that lie at the heart of the phenomenon. Recovery does not mean a cure, but rather an experience of adaptation to symptoms, well-being and a redefinition of personal identity [19,57,58] to someone who was diagnosed with schizophrenia, states that recovery is an attitude towards various possibilities, an active stand and a non-linear process during which a person must find ways to face daily challenges. A person may thus not only try and fail, but may also try again in order to attain personal and professional goals. A relapse is therefore in no way an impediment to the process that characterizes recovery, but represents a move on to another stage.

Qualitative studies have helped shore up and better document the concepts of process and subjective experience [9,11-16,59,60]. Moreover, a number of phenomenological studies have described some conditions that characterize recovery, though without specifying the interaction between them and their impact on people's experience [52,61-66].

The evolution of knowledge about the conditions of recovery has resulted in proposals of a variety of models, whose main emphasis is on services that focus on recovery Anthony [6,18] thus describes the services of a recovery-oriented system by suggesting 12 organizational markers, such as integration and accessibility of services. These markers are interesting and probably useful for a healthcare system seeking to orient services towards recovery, but they are not specific to recovery, and they do not take the subjective nature of the process into account. Fisher and Ahern [20] who began the PACE (*Personal Assistance in Community Existence*) project, put forward a model of recovery that centres on empowerment. It attaches considerable importance to the concept of the power to act and to integrating people into society, but it neglects some essential conditions for recovery, as a number of papers have pointed out. Other investigators [67] have also proposed a preliminary model of recovery as part of a program for following people with a serious mental health problem in the community (*Program for Assertive Community Treatment*). This model has proven particularly useful by highlighting the concepts of engagement and trust and the development of the therapeutic alliance between the care provider and the individual. However, the authors define their model solely from the perspective of the care providers and do not take into account the perspective of the people being followed.

Jacobson and Greenley [4] draw on a synthesis of the literature to put forward a model of practices that group together internal conditions and external ones associated with recovery. In their model, recovery may be positively influenced both by the individual's personal characteristics (internal conditions) and by environmental factors (external conditions). The internal conditions most notably include hope, the meaning given to healing, empowerment, and the capacity to establish and maintain interpersonal relations. The external conditions include respect for human rights and recovery-oriented services. Specifically, respect for human rights means equity in terms of power and resources, as reflected in the right to education, work, housing, and accessible health services. Although the authors maintain that these internal and external conditions are inextricably linked, they do not demonstrate the interplay between these factors as the affect recovery. Moreover, while the internal and external conditions they identify seem, at first sight, to be the ideal ones for facilitating recovery, they do not consider the conditions that hamper recovery and represent the true challenge faced by people trying to recover. More recent studies examine recovery from the standpoint of the role of work in people's life [55] or of rehabilitation services and programs that may foster it [24,67-75]. Carling [76] presents the first model developed in Canada: a frame of reference locating individuals at the centre of services, the purpose of which is to support them in the recovery process. According to this model, the success of recovery depends on the individual and on having a place to live, a paid job and vocational training. The family, groups and associations that individuals surround themselves with are also important "success factors."

### Limitations of the studies

It should perhaps be noted that the body of knowledge on recovery is under construction, given the limitations that can be observed in the numerous studies reviewed [77]. The longitudinal studies deal mainly with the progression of the disease rather than with the phenomenon of recovery. The autobiographical and anecdotal accounts have provided food for thought and shaken beliefs about recovery, but they have not been the subject of systematic research. Furthermore, some may argue that the people who published these documents are different from most people with a mental health problem. The qualitative studies have primarily employed research methods aimed principally at describing the phenomenon of recovery. Those studies that have put forward theoretical models have used very few data sources and are not very explicit about the inclusion criteria for participants, the process of analysis or how the different components of the phenomenon are linked together. The literature reviewed thus does not provide sufficient clarification of the dynamics of recovery; that is, the reciprocal influence of the personal, environmental and organizational conditions that characterize the process. Indeed, the authors of the studies we have reviewed seem to assume that the conditions interact amongst themselves and exert a similar "one-way" effect on all people without distinction as to the type of mental health problem. This state of affairs may reflect the complexity of recovery or the difficulty investigators have in providing multiaxial explanations for the phenomenon that better delineate the operational mechanisms at work between the different conditions involved in the dynamics of recovery. In this regard, the theoretical model put forward in the study by Noiseux [2] constitutes a first step that will make it possible to elucidate the nature of the "two-way" linkages in play.

### Theoretical framework

#### Development of a theoretical model of recovery

The aim of the study by Noiseux [2] was an in-depth understanding and description of the different dimensions of recovery based on the perceptions of three groups of people directly concerned with the phenomenon: people with schizophrenia, family members and care providers. From the analysis, there emerged **seven empirical conditions or indicators **that characterize the recovery process: (a) the experience of schizophrenia, the descent into hell, (b) igniting a spark of hope, (c) a process of introspection, (d) activating the instinct to fight back, (e) discovering keys to well-being, (f) the capacity to manage the unequal interplay between internal and external forces, and (g) seeing light at the end of the tunnel. These conditions comprise specific characteristics or attributes whose reciprocal influence gives rise to a first theoretical explanation of recovery.

Thus, because of the recurrence and persistence of the schizophrenic symptoms that overcome them, people see their life turned upside down and may come to experience a real **descent into hell**. When they hit bottom, and the suffering has become unbearable, they feel a **spark **that leads them to engage in a **process of introspection**. If such incidents or events occur to people who have a **fighting spirit**, they represent conditions that the participants themselves connect with recovery or that analysis of the data allows one to associate with conditions that affect recovery. These conditions lead, in particular, to the **discovery of keys to well-being **that enable individuals to **manage the internal and external forces **and to at last **see light at the end of the tunnel**.

These indicators describe not only the complex, painful subjective reality of living with schizophrenia, but also the daily efforts and great perseverance that enable people to play an active role –or role of "actor"–in their recovery. They thus call into question the traditional, paternalistic practices, which assign the individual a more passive role. The role of family members and care providers then becomes that of a "director" who underscores the individuals' efforts and victories, even the smallest ones; who supports them when they suffer relapse or failure; and who encourages them and helps them find their footing again despite the internal fragility that is under constant threat from internal and external reality.

The participants in the study by Noiseux [2] also agree that recovery does not exclude the possibility they will have symptoms or suffer a relapse. However, having engaged in the process during crisis episodes, they are able to recognize how far they have come and breathe new life into themselves so that they can start again where they left off in learning to live with schizophrenia. Noiseux's [2] findings give rise to a definition of recovery as "a process involving intrinsic, non-linear progress that is primarily generated by the role as actor that the individual adopts to rebuild his or her sense of self and to manage the imbalance between internal and external forces with the objective of charting a path through the social world and regaining a sense of well-being on all psychosocial levels." In the final analysis, it was possible to push the study of recovery beyond descriptive analysis by bringing out the dynamics of the process through a detailed, extensive presentation of the reciprocal influences exerted by the individual, environmental and organizational conditions that characterize it. This theoretical conception of recovery is something new and innovative in the field of mental health in that it offers a vision that differs from the one traditionally associated with the restoration of functional capabilities. In short, the results provide pieces of the puzzle and allow for a better understanding both of the conditions that must obtain for the recovery process to emerge from the individual and a of how care providers can facilitate and sustain these conditions. This process of extending and refining the theoretical model of recovery must be continued; the findings of previous studies must be considered, and, most particularly, a variety of data sources must be used to broaden our understanding of recovery to include people with other types of mental health problem.

## Methods and design

### Research Design

A qualitative, inductive design was selected to develop our understanding of the process of recovery in mental health. By giving prominence to the perspectives of different groups of actors, qualitative research opens the way to learning–from the inside–about the dilemmas and issues people face in their recovery. Given our current state of knowledge and the research questions posed in this study, the methodology chosen was the grounded theory approach. This approach seems most suitable for developing a theoretical model to explain a complex, little-known, ever-evolving process [17]. Indeed, it makes it possible to elicit the perceptual and interactional features of the phenomenon under study from the empirical data and to situate it in a specific social context. Drawing inspiration from symbolic interactionism, grounded theory seeks to reveal the richness of the empirical world by constructing theoretical models that are firmly anchored in reality [26]. According to Paillé [78], the theoretical explanation of a reality under investigation must be faithful to the perspectives and understanding of the people or social actors who give their accounts of it. Furthermore, grounded theory accords particular importance to observed phenomena, which are meaningful insofar as they are expressed in words or social interactions [28]. The investigator's concern is thus not to validate previously selected hypotheses. Rather, it is to engage in a process of recursive analysis in order to understand and interpret the actors' cognitive representations of–or perspective on–their reality and their resulting behaviour [79-81].

### Selection of settings and participants

#### Settings

This multisite study will be conducted in four regions in Quebec: (a) Montreal, (b) Quebec City, (c) Trois-Rivières. We will thus be able to recruit participants from different rural and urban social environments that provide different levels of mental health services. In the four sites, three types of settings will be selected in order to involve a variety of actors or data sources: (a) structured treatment programs administered by a hospital or specialized department of psychiatry (e.g., Louis-H. Lafontaine Hospital, the Pavillon Albert-Prévost at the Sacré-Cœur de Montréal Hospital, the Robert-Giffard Hospital, (b) self-help groups (e.g., Schizophrenia Society of Canada, Depression and Maniaco-Depression Association) and (c) community organizations (e.g., APUR).

#### Participants

The participants selected for this study are people with a mental health problem, a member of their family and one of their care providers. The plan is to select a total of 108 participants, divided into four comparison groups representing respectively the four types of mental health problem under investigation (schizophrenic disorders, affective disorders, anxiety disorders, and borderline personality disorders). Each comparison group (n = 27) will be made up of 12 units. Each unit will comprise one person with a mental health problem, one member of his or her family and one of his or her care providers. This theoretical sample of 108 participants will be divided among the four sites in accordance with population density **(see **Table 1). The composition of the sample means that it can provide a unique opportunity for collecting a wide range of data and thus make it possible to triangulate the perspectives of different people (individuals with a mental health problem, family members, and care providers) who, in one way or another, are involved in an experience of recovery. We should make it clear that the size of the sample is not a function of statistical representativeness. It is based rather on the information that appears necessary to reach empirical saturation, that is, the point at which participants add no new information about the phenomenon being studied. Moreover, constructing the sample in this way makes it possible to organize a complex universe and thus take a crucial step towards developing a substantive theoretical model [27]. In brief, this type of sample offers the possibility of both minimizing and maximizing differences and contrasts in the recovery process because it allows for examination of the phenomenon under different conditions and thus supports exploration of a wide range of variations. Variation is indeed a key tool in grounded theory, for it breaks through narrow specification to broaden the scope of theory. The sample size for our study is not statistically representative, but it was adequate to achieve saturation of the categories. In short, the methodological approach used allows us to triangulate the various perspectives and reach a first stage in theory building. The decisions regarding the sample size of this theoretical and purposeful sample study are based on qualitative research's criteria [82]. One of the principal criteria is that the amount and the depth of data collected (n = 3) is more important to achieve the aims of a qualitative study than the number of participants. Another criteria is saying that triangulation of multiple sources of data (In this study: participants, settings, types of mental health problems), types of participants (people with a mental health problem, n = 12; family members, n = 12; caregivers, n = 12) numerous research tools (n = 4), sites in accordance with population density (n = 3), types of mental health problems (n = 4) are all insuring the rigor of the process and providing strong substantiation of constructs. Another reason has been influencing the decision making on the sample size. This study on recovery model has been inspired from the results obtained and tools elaborated in other studies on the phenomenon that were conducted by the present group of investigators, thus giving room to methodological experience supporting the sample choice [2,5].

**Table 1:** Formation of the theoretical sample

**Sites**	**Types of mental health problems**				
	Schizophrenia	Affective	Anxiety	Borderline personnality	**Total**

Montreal	3 u	3 u	3 u	3 u	12 u

Quebec City	3 u	3 u	3 u	3 u	12 u

Trois-Rivières	3 u	3 u	3 u	3 u	12 u

**Total**	9 u	9 u	9 u	9 u	36 u
	n = 27	n = 27	n = 27	n = 27	**n = 108**

1 u = 1 unit = 1 person with a mental health problem, 1 family member and 1 care provider

**Participants will be selected in accordance with the following inclusion and exclusion criteria: To be included in the study, people with a mental health problem must**: (a) have a diagnosis of schizophrenic disorder, affective disorder, anxiety disorder, or borderline personality disorder based on a psychiatric evaluation; (b) be able to identify a significant family member and a care provider likely to take part in the study; (c) live in the community; (d) be in a stable condition (able to manage symptoms) and (e) know how to speak, read and write French. **To be included, family members must**: (a) have a significant bond with the person with the mental health problem who has agreed to take part in the study (e.g., parent, spouse, brother, sister, friend) and (b) know how to speak, read and write French. **To be included, care providers must**: (a) have followed a person with a mental health problem who has agreed to take part in the study for at least the past year (nurse, psychiatrist, social worker, psychologist, etc.) and (b) know how to speak, read and write French.

**People with a mental health problem and their family members are excluded if they**: (a) go through an acute crisis episode and are hospitalized; (b) have a major physical health problem; or (c) are diagnosed as intellectually deficient.

**Recruitment of participants**: Different strategies will be used to select the participants in the settings and sites mentioned earlier. In the hospitals, the research project will be presented to the respective psychiatric departments. Eligible individuals will be identified with the assistance of the heads of client programs (for schizophrenic, affective, anxiety, and borderline personality disorders) in order to enlist their cooperation in recruiting participants who meet the inclusion criteria. The client-program heads will contact the people with a mental health problem who have been identified as eligible to pave the way for a preliminary contact with the investigator to explain the purpose of the study. After the preliminary contact, the individuals will be asked to get in touch with the investigator to say whether or not they are interested in taking part and to confirm the participation of a family member and a care provider, who will also be contacted so that they can be given information on the research project and give their consent to take part.

In the self-help and community groups, the investigator will get in touch with the people in charge to present the research project and prepare a document to publicize the study and ask individuals who might be interested to take part. These individuals will have to contact the investigator or the research assistant directly to obtain information, decide whether they are willing to participate and, if they are, to set a time and place for the interview. Those wishing to take part will also have to identify a family member and a care provider from their network, depending on each case. Every participant will be met for a single interview lasting some 60 to 90 minutes. The interview will be recorded on digital audiotape. The interviewer will have to have professional clinical experience in the mental-health field and will be trained to conduct this type of interview, which necessarily entails creating a climate of trust. The individuals with a mental health problem and their family member will receive monetary compensation for travel and for taking part in the study.

#### Conduct of the study

##### Data collection

Semi-structured interviews are a powerful tool and are considered to be indispensable to an in-depth exploration of the participants' perspectives on the recovery process [83]. Three interview guides will be drawn up: one for the people with a mental health problem, one for the family members and another for the care providers. The interview guides will be developed by researchers and collaborators on the research team who will draw on the interview guides developed in the study by Noiseux [2]. The interview guides will comprise general and specific questions. The general questions will first be formulated in broad terms to encourage participants to express themselves freely on their notion of recovery. The formulation and presentation of the questions will allow for some flexibility, and the interviewers will be able to adjust them depending on how the interview develops with each participant. Then, specific sub-questions on the order of "Who?", "What else?", "How?", and "When?" will be added. The purpose of the questions is, on the one hand, to get participants to elaborate on their perspective on recovery and, on the other hand, to explore more specific themes, such as personal conditions (e.g., sources of motivation, strategies of action, personal resources), environmental conditions (e.g., interpersonal relations, social roles) and organizational conditions (e.g., accessibility of services, healthcare practices) that help or hinder recovery. In addition, sociodemographic and clinical information (e.g., taking a medication) will be collected using a special form (see Appendix 2). Furthermore, a pre-test of the three interview guides will be conducted with 12 people, that is, four people representing the types of mental health problem, four family members and four care providers. The guides will be adjusted, as necessary.

Throughout the study, the interviewers will make field notes to record their observations chronologically. These notes will be used to describe the environment surrounding the interviews, factual events, individuals' reactions, and personal impressions and reflections. The contents of the notes will be shared with the research-team investigators and collaborators in order to discuss possible biases or information that may be relevant to the data-analysis process [84].

##### Data-analysis

Data analysis and data collection will be carried out concurrently, in accordance with the grounded theory approach [17]. The interviews will be transcribed in full and read over systematically in order to carefully review the contents and, if need be, refine the interview questions to ensure we delineate and understand the phenomenon under investigation. The process of analysis requires constantly moving back and forth between the data collected, the existing literature and the emerging theoretical model. This recursive analysis will be carried out in accordance with the paradigm model Strauss & Corbin [17] which entails three coding procedures: (A) open coding, (B) axial coding and (C) selective coding. Each of these steps will be carried out at the same time for each of the four comparison groups.

###### A) Open coding

The significant events, facts or incidents will be underlined in the transcripts, and themes or keywords corresponding to each of the facts raised by the participants will be noted in the margins. Examples of such themes are relapse, strategy, fight, motivation, and marker. **This first step **may be described as a systematic exploration of the data on the lookout for "chance" discoveries. Once identified, as suggested by Huberman & Miles [84], the themes will be grouped together in individual tables to provide a general portrait of the contents of each interview. **Second**, all the transcribed interviews will be transferred to the data-processing software NU-DIST Vivo (*Nonnumerical Unstructured Data Indexing Searching and Theorizing*) to store and code the data and create a preliminary open-coding grid. The initial codes are word descriptors deduced from the research question or derived by induction directly from the empirical data. At this stage, similar themes emerging from each interview are organized in accordance with the various codes, marking the beginning of the data-interpretation process, as the initial codes are grouped together by properties or characteristics. **Third**, to reach a higher level of conceptualization, related codes will be grouped together to make up categories or concepts. Furthermore, the codes and categories will be subject to continual comparison with the aim of grouping together codes for similar statements, defining categories and bringing out relationships between the categories. These categories will be reviewed and compared, entailing continual movement back and forth between items of empirical data. The grouping of similar codes will undergo numerous changes resulting in numerous versions of the initial open-coding grid. This first stage in the conceptualization process will be submitted to the research-team investigators and collaborators to validate and refine the categories and their properties.

###### B) Axial coding

That operation involves refining the open-coding grid in order to delimit the theoretical model. The categories delimiting the phenomenon under investigation are related to each other in terms of different levels of "conditions": causal conditions, interactional and structural contexts, action strategies, and consequences. The identification of these conditions serves as a point of departure for attaining a better understanding of and delineating the interplay of the individual, environmental and organizational conditions that influence recovery.

###### C) Selective coding

The purpose of the third step in analyzing the data is to integrate and refine the theoretical model. **Three stages are involved. The first **entails a process of integration for each comparison group through the statement of a core category: that is, a few words, a sentence or a conceptual idea that goes to the heart of the phenomenon. In other words, a category is a core category insofar as the other categories can be grafted on to it and variations between them are justified by reference to it [17]. This stage will thus yield four core categories. At the end of the analysis, it will be possible to identify a unifying theme (*selective coding*) of the recovery process for each of the mental health problems or comparison groups. **In the second stage **the model is refined; the aim is to use the four core categories to develop a proposal for a single core category and thus clearly define a theoretical model with generalizable parameters that nonetheless takes into account the particular traits of individuals and their health condition. **To that end**, a discriminant sample will be selected by choosing individuals most likely to enable us to refine the theoretical model [17]. In practical terms, 24 participants will be selected from the 108 people in the initial theoretical sample: that is, two units (n = 6; 2 people with a mental health problem, 2 family members, 2 care providers) per comparison group or type of mental health problem. The 24 participants will form two discussion groups to validate the proposed theoretical model. To obtain as rich a data set as possible, recommend choosing people who can express themselves more easily on the subject under investigation [84].

### Criteria of scientific rigour in qualitative research

Like other qualitative research methods, grounded theory meets the criteria of scientific rigour: credibility, transferability, internal consistency, and reliability.

**Credibility**: This criterion entails data authenticity and that conclusion or propositions respect the data that has been collected [85]. To ensure credibility, the research-team investigators and collaborators will return continually to the empirical data to back up the organization and interpretation of the data. More specifically, field notes will be made and the data transcribed in full as the study progresses, thus increasing its credibility. In addition, continuous rereading of the transcripts, constant revision of the coding by comparing empirical data and meticulous analysis of the data will enhance the authenticity of the findings. **Transferability**: Respect for the principles of theoretical sampling and empirical data saturation will make transfer of the conclusions to settings similar to the one under investigation possible [86]. The detailed description of the settings in which the research is conducted will help determine whether or not the conclusions can be transferred. **Internal consistency**: This criterion relates to the quality of the description of the analytic process [87]. Triangulation with different data-collection tools makes it possible to track and follow the logic of decisions that are made [87] his criterion can be met by reference to the interviews, the field notes, the systematic and rigorous organization of the data, and the open-coding grids of the recovery process. **Reliability**: This criterion will be respected by virtue of providing a transparent explanation of the methodology used in analyzing the data, a detailed description of the people providing the data and validation of the categories and their properties by a discriminant sample of 12 participants selected from the initial 108.

### Timetable

The following is the timetable planned for the project: **September to December 2006**: Development of research tools: guides for the analysis of relevant documents; interview guides; presentation of the project at the four selected sites to the participating groups and organizations. **January to March 2007**: Conduct of pre-tests with 12 people and adjustments to the interview guides. **April 2007 to April 2008**: Beginning of recruitment in the four sites, data collection (interviews) and data analysis, carried out concurrently. A 12-month recruitment period seems appropriate to us, given the specific inclusion criteria requiring a relationship between participants (individual-family member-care provider) to make up a unit. **May to December 2008**: Continuation of data analysis; holding two focus groups to validate the theoretical model of recovery. **January to November 2009**: Finalization of the analysis and the research report; dissemination and circulation of the findings.

### Originality and benefits of the study

The unique and innovative nature of this research project derives from four distinctive features: (a) the background of the research team providing for a "triangulation" of perspectives; (b) the variety of groups, organizations and regions, making it possible to examine the issue in a variety of contexts; (c) the formation of units comprising three data sources; and (d) the continuation of a theory-building process, broadening the explanation of recovery to include four types of mental health problem. In addition, the study will generate an exceptional quantity and quality of qualitative data, from which empirical indicators of the recovery process will be derived. The ultimate goal is to construct a strong knowledge base so that we can clearly define a theoretical model with generalizable parameters that nonetheless takes into account the particular traits of individuals and their health condition. This study has undoubted potential to advance knowledge in the field of mental health. By emphasizing individuals' potential and resources rather than the deficits associated with their disease, the study will probably call some traditional theoretical positions into question. Finally, the heuristic value of a substantive theoretical model should influence policy orientations and the organization of mental health services that really focus on the recovery process.

### Knowledge transfer and strategies for the dissemination of findings

For this study, knowledge transfer is of major importance because the raison d'être of a substantive theoretical model of recovery is to buttress and renew practices for the care of clients living with a mental health problem. To disseminate our findings and promote their incorporation into practice, our knowledge transfer activities should involve more than simple one-way distribution. They should also be based on reciprocal interaction between the investigators and partners from different groups and organizations. Moreover, we plan to bring out a plain-language version of the findings for care providers and self-help associations. Scientific articles reporting the findings will be submitted to various national and international periodicals.

### Ethical considerations

The proposed research project will have to be approved by the scientific and Ethics Committees for Research of the CHUM (SL 06.055), HSCM (C.E. 2006-12-78), CHRTR (CÉR-2006-139-01) and CTR de Nemours (Quebec) that provide services to the community. Potential participants will be given clear explanations of the objective of the research and of the way the interviews are to be conducted. They will then be in a position to give their free and informed consent both to taking part in the study and to having the interviews digitally recorded. All recordings will be kept under lock and key in a secure location at the research centre involved throughout the course of data collection and analysis. Because of the need to disseminate the findings, the recordings will be destroyed seven years after completion of the study. The participants will also be assured of the confidentiality of any identifiable personal information through the use of pseudonyms to prevent recognition. If they suffer discomfort during the interview, participants will be told where they can find help. All participants will be informed of their right to withdraw from the study should they wish to do so.

## Competing interests

The authors declare that they have no competing interests.

## Authors' contributions

The authors approved the manuscript and are taking responsibilities for appropriate portions of the content. SN has made substantial contributions to conception and design, acquisition of data, analysis and interpretation of data and general supervision of the research group. DSCT has made important contribution to revising it critically for important intellectual content, analysis and interpretation of data. CL has been involved in the proposed research project will have to be approved by the scientific and Ethic Committee for CHRTR and his analysis data. NR has made a contribution to conception and design in this study protocol. RM has made important contribution to revising it critically for important intellectual and clinical content. EC has been a major contribution in analysis and interpretation of data. Finally, RL has been involved in the analysis and interpretation of data. What is more, the very fact these authors are being brought together from different sites and settings will facilitate participant recruitment. All authors read and approved the final manuscript.

## Pre-publication history

The pre-publication history for this paper can be accessed here:


